# Numerical and experimental methods used to evaluate active drag in swimming: A systematic narrative review

**DOI:** 10.3389/fphys.2022.938658

**Published:** 2022-10-20

**Authors:** Tiago J. Lopes, Jorge E. Morais, Mafalda P. Pinto, Daniel A. Marinho

**Affiliations:** ^1^ Department of Sport Sciences, University of Beira Interior, Covilhã, Portugal; ^2^ Research Center in Sports Health and Human Development (CIDESD), University of Beira Interior, Covilhã, Portugal; ^3^ Department of Sport Sciences, Instituto Politécnico de Bragança, Bragança, Portugal

**Keywords:** active drag, water resistance, biomechanics, assisted swimming, resisted swimming

## Abstract

**Introduction:** In swimming, it is necessary to understand and identify the main factors that are important to reduce active drag and, consequently, improve the performance of swimmers. However, there is no up-to-date review in the literature clarifying this topic. Thus, a systematic narrative review was performed to update the body of knowledge on active drag in swimming through numerical and experimental methods.

**Methods:** To determine and identify the most relevant studies for this review, the Preferred Reporting Items for Systematic Reviews and Meta-Analyses (PRISMA) approach was used.

**Results:** 75 studies related to active drag in swimming and the methodologies applied to study them were analyzed and kept for synthesis. The included studies showed a high-quality score by the Delphi scale (mean score was 5.85 ± 0.38). Active drag was included in seven studies through numerical methods and 68 through experimental methods. In both methods used by the authors to determine the drag, it can be concluded that the frontal surface area plays a fundamental role. Additionally, the technique seems to be a determining factor in reducing the drag force and increasing the propulsive force. Drag tends to increase with speed and frontal surface area, being greater in adults than in children due to body density factors and high levels of speed. However, the coefficient of drag decreases as the technical efficiency of swimming increases (i.e., the best swimmers (the fastest or most efficient) are those with the best drag and swimming hydrodynamics efficiency).

**Conclusion:** Active drag was studied through numerical and experimental methods. There are significantly fewer numerical studies than experimental ones. This is because active drag, as a dynamical phenomenon, is too complex to be studied numerically. Drag is greater in adults than in children and greater in men than in women across all age groups. The study of drag is increasingly essential to collaborate with coaches in the process of understanding the fundamental patterns of movement biomechanics to achieve the best performance in swimming. Although most agree with these findings, there is disagreement in some studies, especially when it is difficult to define competitive level and age. The disagreement concerns three main aspects: *1*) period of the studies and improvement of methodologies; *2*) discrimination of methodologies between factors observed in numerical vs. experimental methods; *3*) evidence that drag tends to be non-linear and depends on personal, technical, and stylistic factors. Based on the complexity of active drag, the study of this phenomenon must continue to improve swimming performance.

## Introduction

Swimming performance concerning humans is poor compared to species whose habitat is aquatic. In fact, the maximum swimming speed performed by humans represents about 16% of the maximum speed obtained by aquatic species (Toussaint et al., 2004). One of the reasons for this difference in speed is the greater resistance humans encounter when moving through the water (Toussaint et al., 2004).

A swimmer’s displacement relies on the net balance between propulsion and drag (Zamparo et al., 2020):

$$a=\frac{T-D}{m}$$ (1)

In which a is the acceleration (in m/s^2^), T is the total propulsive force, i.e., thrust (in N), D is the total drag force (in N), and m is the total mass (i.e., swimmer’s body mass plus the added mass of water, in kg). This is critical to understanding the biomechanical needs that determine better swimming performance. Therefore, when performing swimming strokes, the goal is to optimize speed by increasing propulsion and reducing drag (Zamparo et al., 2020). Drag is the force that swimmers must overcome to maintain the translation of their center of mass (Kjendlie and Stallman, 2008). It can be expressed by Newton’s equation as:

$$D=\frac{1}{2}∙v^{2}∙\rho ∙S∙C_{d}$$ (2)

In which D is the drag force (in N), ρ is the density of water (in kg/m^3^), v is the swimming speed (in m/s), S is the projected frontal surface area (FSA) of the swimmers (in m^2^) and C_d_ is the coefficient of drag (changing according to shape, orientation, and Reynolds number).

The total drag consists of three components: *1*) friction drag (depends on the friction between the skin and the water); *2*) pressure drag (depends on body surface area); *3*) wave drag (depends on the water surface deformation) (Toussaint and Beek, 1992). Based on these components, total drag can be computed as:

$$F=F_{f}+F_{p}+F_{w}$$ (3)

In which F (in N) is the total drag force, F_f_ is the friction component (in N), F_p_ is the pressure component (in N), and F_w_ is the wave component (in N). Overall, it is generally accepted that frictional drag is the component with the smallest contribution to total drag, especially at higher swimming velocities (Bixler et al., 2007). Nonetheless, friction drag should not be disregarded in elite level swimmers. On the other hand, pressure drag and wave drag represent the most important part of the total drag, especially when performing a swimming stroke (Toussaint and Beek, 1992). Therefore, swimmers must intensify the most hydrodynamic postures during swimming.

Indeed, the literature reports two types of drag: *1*) passive drag; *2*) active drag. Passive drag (D_p_) is the evaluation of the drag produced during the displacement of a towed body (i.e., without relative movement of the body segments in the aquatic environment) (Pendergast et al., 2006). Active drag (D_a_) is the water resistance induced to a body while swimming (Kolmogorov and Duplishcheva, 1992). Studies on D_a_ are more common because during a race swimmers spend most of their time performing strokes (Morais et al., 2019).

In 1974, di Prampero et al., developed and used a method to evaluate drag during real swimming conditions through an energetic approach. All recent overviews of a swimmer’s drag have confirmed this statement (Keys and Lyttle, 2007; Sacilotto et al., 2014; Morais et al., 2019; Zamparo et al., 2020; González-Ravé et al., 2022). Both types of drag and its components can be measured by numerical and experimental methods. The former (i.e., numerical methods) is a virtual prototype of the product of interest represented by a system of equations based on a mathematical theory, such as computational fluid dynamics (CFD) (Takagi et al., 2016). The latter (i.e., experimental methods) is a method in which the variables are manipulated in a pre-established way and their effects are sufficiently controlled and known by the researcher for the observation of the study (Takagi et al., 2016). CFD is one of several methods that have been applied in sports research to observe and understand the water flow activity around the human body and its application to improve swimming technique, equipment, and performance (Keys and Lyttle, 2007; Marinho et al., 2011). Smooth particle hydrodynamics (SPH) is a numerical method without a Lagrangian mesh, which allows a detailed quantitative analysis of swimming stroke variations and kinanthropometric variations. It is important to mention that there are few studies that use numerical methods to study D_a_. Bixler and Schloder (1996) introduced two-dimensional CFD applied to swimming science. More recently, Cohen et al. have made progress in this method as they are the authors of some studies on numerical methodology that provide some interesting data (Cohen et al., 2015; Cohen et al., 2018; Cohen et al., 2020). However, in one of their studies, they mention that the angles of attack of the hands were compared with the contribution of lifting and dragging the hands to generate thrust in the direction of the current. This study allowed the investigation of possible connections between performance and asymmetries during swimming. Efficiency is negatively affected because periods of very high velocity consume exaggerated amounts of energy, considering that drag is non-linearly dependent on instantaneous velocity. Thus, a greater coefficient of variation of the swimmer’s speed suggests a lower swimming efficiency (Cohen et al., 2018; Cohen et al., 2020).

Based on experimental methods, D_a_ can be measured through three approaches: *1*) measurement of active drag (MAD) (Hollander et al., 1986); *2*) velocity perturbation method (VPM) (Kolmogorov and Duplishcheva, 1992); *3*) assisted towing method (ATM) (Alcock and Mason 2007), and; *4*) measurement of residual thrust (MRT) (Narita et al., 2017). To determine D_a_ through experimental studies, it was found that MAD, VPM, and ATM are now commonly used to obtain D_a_ values accurately to assess swimmer technique (Toussaint et al., 2004; Formosa et al., 2012; Hazrati et al., 2016). The MAD system consists of pushing pads while the swimmer moves in the water performing the natural swimming movement (as much as possible) (Hollander et al., 1986). The thrust pads fixed below the water allow for the generation of propulsion without loss of energy (Formosa et al., 2012). The ATM system is relatively new compared to the MAD and VPM systems (Hazrati et al., 2016). The ATM system was developed identically to the bases of the VPM, except that it uses assisted towing and resisted swimming (Toussaint et al., 2004), as similar conditions are required in both tests. The main difference between the two is that the ATM produces D_a_ profiles and intra-course propulsion, rather than just an average measure of D_a_ (Formosa et al., 2012). The MRT method, which was recently developed, allows the estimation of drag in swimming using measured values of residual thrust (Narita et al., 2017; Narita et al., 2018b; Gonjo et al., 2020). Through this method, it is possible to investigate D_a_ at various speeds without neglecting the influence of stroke length.

As stated by Toussaint et al., 2004, it is known that human performance in water is dependent on many variables in addition to innate ones. In this way, we must consider all the variables that can compromise a better performance. Thus, these variables depend not only on their propulsive abilities but also on their ability to reduce to a minimum the drag forces that involve the body in a hydrodynamic way (Taïar et al., 1999). Studying active drag becomes relevant simply because it corresponds to the very act of swimming in a cyclical way, which consists almost of the entire race in high competition (Kolmogorov and Duplishcheva, 1992). Considering the importance that the measurement of drag has on swimming performance, it can be said that the evidence in the literature has not been systematically or narratively summarized, specially including studies based on both numerical and experimental measurements. It must be mentioned that Sacilotto et al. (2014) underwent a literature review on drag that also included numerical studies. The authors performed a biomechanical review of the techniques used to estimate or measure resistive forces in swimming. Therefore, the aim of this study was to carry out a systematic narrative review focusing on D_a_ (and its components) measured by numerical and experimental methods.

## Methods

### Literature search and article selection

**FIGURE 1:**
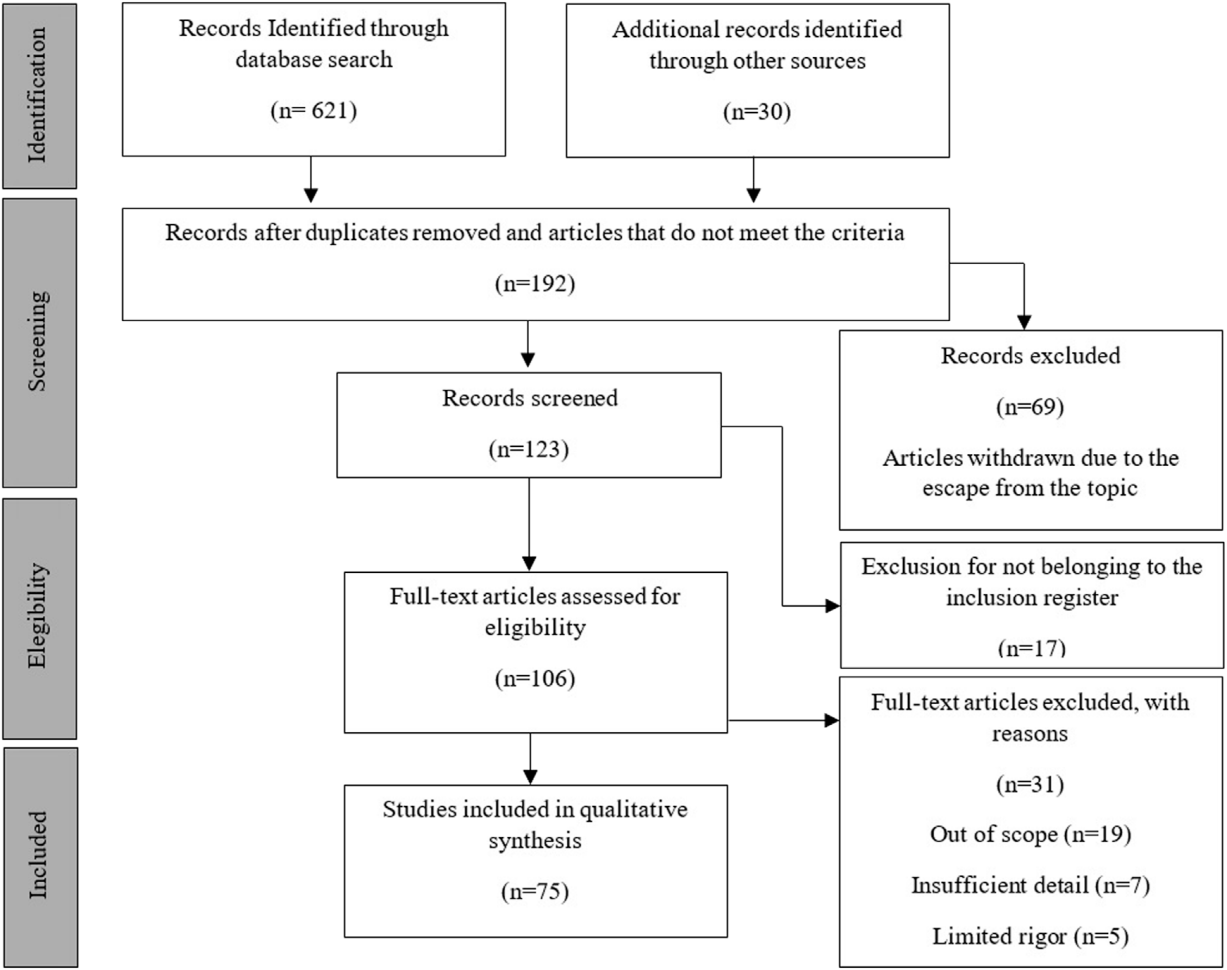
Summary of PRISMA flow for search strategy.

Studies that analyzed D_a_ in swimming were searched in the following databases: Web of Science, Scopus, PubMed, and Science Direct. These electronic search databases were chosen as the most common databases related to methodological approaches in biomechanics applied to sport (framework, methodology, performance, and engineering). The studies that were selected met the following pre-defined inclusion criteria: *1*) follow the criteria defined in Table 1; *2*) are observational or intervention studies, *3*) are written in English, *4*) are published in a peer-reviewed journal; *5*) involve fully healthy real human swimmers (or their three dimensional scans – 3D); *6*) include tests performed to determine D_a_ in swimming; *7*) are related to the analysis of human movement in the aquatic environment; *8*) use numerical and experimental methods. Review articles, conference articles and books, studies including animals, and publications not related to the topic in question were excluded from the analysis. Studies with disabled swimmers were also excluded from this review. The Preferred Reporting Items for Systematic Reviews flow diagram (PRISMA in Figure 1) characterizes the identification, screening, verification of eligibility, and inclusion of the studies. PRISMA describes the flow of information through the different phases of a systematic review and includes maps or number of identified, included, and excluded records and reasons for exclusion.

**TABLE 1:** PI(E)CO (P – patient, problem or population; I – intervention; E – exposure; C – comparison, control, or comparator; O – outcomes) search strategy.

Population	Intervention or exposure	Comparison (design)	Outcome
Swimmer^a^	Development	Cross-sectional	Active drag
Athlete^a^	Long-term development	Longitudinal	Drag
Boy^a^	Biomechanics	Experimental	Performance
Girl^a^	Strength and conditioning	Descriptive	Coefficient of drag
Young^a^	Performance	Randomized control trial	Mechanical power
Men^a^	Competitive	Numerical	Assisted swimming
Women^a^		CFD	Resisted swimming
Male^a^		MAD-System	Forces
Female^a^		VPM	Drag forces
		Computational fluid dynamics	Biomechanic
		Quantitative analysis	Power input
		ATM	Power output
			Mechanical
			Water resistance
			Coefficient
			Friction
			Inverse dynamics
			Posture
			Hydrodynamic
			Resistance
			Balanced position
			Alternative fluid dynamic
			Underwater
			Body position
			Breaststroke
			Backstroke
			Front crawl
			Freestyle
			Butterfly
			Balance

^a^ truncation to retrieve words with different endings.

The Patient/Problem, Intervention, Comparison and Outcome (PICO) search strategy is shown in Table 1. It presents the words used to carry out the research, supported by the words most used by the authors to describe their studies. Each title, abstract, and keyword field of the text was identified and carefully read for the first selection of journal articles. If any of these fields (title, abstract, and keywords) was not clear on the topic under analysis, it was necessary to read and review the entire article in question to ensure its inclusion. For the initial research, a Boolean search strategy was used based on a combination of keywords that can be seen in Table 1. After excluding all unrelated and duplicate articles, 75 articles were selected for the final published review (Figure 1), comprising studies from 1986 until the end of the review research on 31 January 2022, as this was the latest study framed within the pre-defined selection model. From the selected articles, the reviewers extracted information about the aim of the study, the participants, the methods to measure the D_a_ in swimming, the characteristics of the numerical and experimental method (s), the measured variables, and the data analysis used.

### Quality assessment

The Delphi method was used to assess the quality of the selected articles (knowing that Delphi is a process to develop a scale suitable for the purpose). It was noted that this approach (i.e., applying and creating a group scale) is an indicator of methodological quality (de Meyrick, 2003; de Morton, 2009). The Delphi method aims to structure a process of collective communication allowing a group of researchers to deal with a complex problem (de Meyrick, 2003). This method allows the creation of an evaluation scale for the articles selected for this study (de Meyrick, 2003; de Morton, 2009). Particularly when accessing numerical studies, there is a need to create a specific questionnaire and scale. Thus, it was agreed among the authors to create a questionnaire that would make the decision on the classification of the studies selected for this narrative review unanimous. In this way, through the Delphi method, the authors attempted to evaluate the following questions: *1*) Does the contemplated content meet the objective?; *2*) Was there a logic in the used methods?; *3*) Were the methods and subjects well defined?; *4*) Was there writing, language and clarity in the presentation of the contents covered?; *5*) Was the presentation of the results clear?; *6*) Are the results consistent with the culture of the study? Two independent reviewers read all articles and scored according to the items on the scale (poor quality if scored ≤2; fair quality if scored 3 to 4; high quality if scored 5–6) (de Meyrick, 2003; de Morton, 2009). Subsequently, Cohen’s Kappa (K) was calculated to assess agreement between reviewers. It was interpreted as *1*) no agreement if K ≤ 0; *2*) none to slight if 0.01 < K ≤ 0.20; *3*) fair if 0.21 < K ≤ 0.40; *4*) moderate if 0.41 < K ≤ 0.60; *5*) substantial if 0.61 < K ≤ 0.80; *6*) almost perfect if 0.81 < K ≤ 1.00 (McHugh, 2012). After reviewing all articles, the Delphi scale showed a mean score of 5.85 ± 0.38 (i.e., high quality if scored), and Cohen’s Kappa an almost perfect agreement between reviewers (K = 0.651, *p* < 0.001). The Delphi scores are presented in Table 2 for each article.

**TABLE 2:** Summary of the objective, sample demographics, and main results of the studies related with D_a_ for numerical methods.

Study (year)	Objective	Subjects (age and competitive level)	Results	Delphi score Mean ± 1 SD
Cohen et al. (2012)	Determine the relative importance of the extension kick (often called downbeat) compared to the flexion kick (often called upbeat) in dolphin kick swimming	Smoothed Particle Hydrodynamics (SPH). Laser scans of athletes are used to provide realistic swimmer geometries in a single anatomical pose. These are rigged and animated to closely match side-on video footage	Swimmer strength depends on kick frequency and is insensitive to ankle flexibility. The maximal drag force occurs in the direction of the current, corresponding to the periods before the inversions of strokes, and swimmers must pay attention to the rapid inversions of direction	5.0 ± 0.0
Cohen et al. (2014)	Determine the pitching effects of buoyancy during all competitive swimming strokes (front crawl, backstroke, butterfly, and breaststroke)	Laser body scans of national-level athletes and synchronized multiangle swimming footage were used in a novel markerless motion capture process to produce three-dimensional biomechanical models of the swimming athletes	Variation in buoyancy torque is much larger during breaststroke and butterfly than during front crawl and backstroke; pitching swimmer moment of inertia varies much more for butterfly and breaststroke than for front crawl and backstroke; that buoyancy torque and pitching swimmer moment of inertia are anticorrelated during butterfly and breaststroke	5.0 ± 00
Cohen et al. (2015)	A combination of kinematic data and SPH-based flow modeling was used to explore the degree to which the instantaneous impulse generated by the arms is controlled by the trajectories of the hands, their orientation and speeds during the front crawl stroke	SPH fluid model is used to analyze the thrust and drag generation of a front crawl swimmer. The swimmer model was generated using a three-dimensional laser body scan of the athlete and digitization of multi-angle video footage (CFD)	Two large distinct peaks in liquid thrust coincide with underwater strokes. The movement of the hands generates vortex structures that travel along the body (there is the production of lift and drag)	6.0 ± 0.0
Cohen et al. (2018)	Investigate how the streamwise speed and net streamwise forces of the swimmer vary throughout the phases of the stroke. The dependence of the relative thrust from the arms compared to the legs on the stroke rate was also investigated	A dynamic biomechanical model of a female national-level swimmer was generated from a three-dimensional laser body scan of the athlete and multi-angle videos of sub-maximal swimming trials (CFD)	The Froude number varies from 0.40 to 0.31, meaning that the swimmer swims close to Fr = 0.42 (hull speed), consequently the drag of waves on the surface is significant	6.0 ± 0.0
Cohen et al. (2020)	The asymmetrical front crawl swimming performance of a male elite level swimmer who breathed every second arm stroke (unilaterally) was investigated	A laser body scan and multi-angle video footage of the athlete were used to generate a swimming biomechanical model (one male elite level)	The natural asymmetrical performance with the swimming movement acquired through the frontal area results in a greater D_a_ (swimmer’s technique) are the main findings. These will help improve athletes’ performance and coaches’ decision making	6.0 ± 0.0
Keys and Lyttle, (2007)	Sought to discriminate between the D_a_ and propulsive forces generated in underwater dolphin and flutter kicking using the CFD technology	A 3D image of an elite swimmer was animated using results from a kinematic analysis of the swimmer performing two different patterns of underwater dolphin kick (large/slow kicks *versus* small/fast kicks) and the underwater flutter kick	Advantage in using the swim kick in the underwater flutter kick over the small/fast or large/slow kick at 2.18 m/s There are benefits in prescribing techniques through the use of CFD models	6.0 ± 0.0
Yuan et al. (2019)	Find the mechanism of the hydrodynamic interaction between human swimmers and to quantify this interactive effect by using a steady potential flow solver	Only interested in the wave drag component. No attempt is made here to analyze the other drag components due to the viscosity of the fluid	Showed that the hydrodynamic interaction made a significant contribution to the drafter’s wave drag. By following a leading swimmer, a drafter at wave-riding positions could save up to 63% of their wave drag at speed of 2.0 m/s and lateral separation of 2.0 m/s. When a drafter is following two side-by-side leaders, the drag reduction could even be doubled	5.0 ± 00
		One passive swimmer; three swimmers in competitive swimming; and another observations		

D_a_, active drag; CFD, computational fluid dynamics; SPH, coupled biomechanical-smoothed particle hydrodynamics; F_r_, froude number; C_d_, coefficient of drag; SD, one standard deviation.

## Results

A total of 75 studies met the inclusion criteria, of which seven used the numerical method and 68 the experimental method. The criterion for defining which studies to include was unanimous, and so it was decided to consider all studies regardless of their type of method. However, it was essential that the topic of the study followed the needs described in Table 1.

Table 2 present a summary of the included studies, indicating the authors, year of publication, objective, number of participants, if applicable, and main results, for studies based on numerical and experimental methods, respectively.

Seven studies analyzed D_a_ based on numerical methods (including five studies on front crawl, two on backstroke, four on butterfly, and one on breaststroke (considering front crawl and dolphin kick), in which some studies include several techniques) (Table 2). All studies used swimmers as a sample, despite being models (scans of athletes or three-dimensional programming of at least one or more swimmers as their sample). Sixty-eight studies analyzed D_a_ based on experimental methods (including 64 studies on front crawl, six on backstroke, two on butterfly, and six studies on breaststroke (considering front crawl and dolphin kick)) (Supplementary Table S1). They used human swimmers in their entire sample, all with effective experience in the modality and training.

## Discussion

The aim of this study was to perform a systematic narrative review on the up-to-date body of knowledge on D_a_ and its components through numerical and experimental methods. In the studies that used numerical methods for the D_a_ analysis, it was found that the main focus was to: *1*) confirm whether the drag measured the same force throughout the entire path; *2*) verify the variation within the stroke cycle or between stroke cycles. Overall, the studies that focused on the experimental methods to assess D_a_ tended to: *1*) present the comparison between the determining factors of performance; *2*) emphasize the comparison between the drag variation at different swimming speeds and between sexes and age groups.

### Numerical methods – D_a_

Most studies were focused on the front crawl stroke for submaximal speeds (Cohen et al., 2012; Cohen et al., 2018; Yuan et al., 2019). It was found that the ratio of arm thrust to leg thrust increases with a higher stroke rate (Cohen et al., 2018). However, the attempt at specificity is also evident, i.e., they investigate specific movements such as kicks and arm strokes (cycles). Another study also analyzed the effects of buoyancy during swimming and the drafting as a parameter performance for competition (Cohen et al., 2014). Additionally, the authors observed that at different flow velocities, the hydrodynamic coefficients considered were not constant, knowing that the variation for different hand positions was examined for different phases of the path (Cohen et al., 2018). Regarding D_a_ using numerical methods (Table 2), it was noted that the coefficient of variation (CoV) decreases from 4.8% at the lowest frequency to 3.9% at the highest frequency, indicating that velocity fluctuations decrease with the stroke rate (Cohen et al., 2018). It also highlights the asymmetries in the duration of the different phases of the strokes. The right arm had a 33% shorter impulse period and a 14% longer recovery period than the corresponding periods of the left arm. The duration of the traction phase was similar for both arms (Cohen et al., 2018). There are differences between the use of the underwater flutter kick over the large/slow kick or small/fast kick at 2.18 m/s (Keys and Lyttle, 2007), confirming that the D_a_, in relation to all these variables, is entirely influenced by the great variation between asymmetries, type of stroke, type of kick, and considering the type of style the swimmer is performing.

During the movements, vortex structures are generated by the arms, which then pass along the body towards the movement of the legs (using a female swimmer at submaximal speed in front crawl). These structures dissipate quickly due to the high-frequency kicking of the legs. There are earlier and more recent references (Cohen et al., 2018; Cohen et al., 2020) that suggest that generated vortices can be used to increase propulsion through vortex recapture. Another study that determined the pitching effects of buoyancy during all competitive swimming strokes (front crawl, backstroke, breaststroke, and butterfly) with a male swimmer and a female swimmer at constant submaximal speed verified that the average thrust torque tended to increase in the legs and decrease in the head (Cohen et al., 2014). However, the instantaneous torque had an opposite effect during part of the throttle stroke. In addition, the alternating techniques (front crawl and backstroke) showed smaller variations in the positions of the center of mass, thrust torques and positions of the center of thrust (Cohen et al., 2014). The simultaneous techniques (butterfly and breaststroke) showed greater variations in buoyancy torques, directly influencing the swimmer’s ability to maintain a horizontal inclination to perform the strokes. This helps athletes swim efficiently by minimizing their frontal areas and the consequent pressure drag (Cohen et al., 2014). The CoV values were moderate for front crawl (53% for women and 26% for men, respectively, this order will be used from now on) and backstroke (52% and 28%), with female values being approximately twice than those of males. The CoV values for butterfly (132% and 133%) and breaststroke (130% and 127%) were significantly higher than for the other strokes. The CoV is higher for strokes with synchronized limb movement. The CoV, and consequently D_a_, change depending on the movement or swimming phase, being different between kicks and strokes (Cohen et al., 2015). Regarding dolphin kicks, it was observed that the CoV of swimming velocity remains small (7–9%) in experiments with dolphin kicks even when the frequency increases. The amplitude of velocity fluctuations increases, which turns out to be much lower than other intracycle simulations (28–59%). The extension kick proved to be more important than the flexion kick (extension can also be known as down in prone swim and up in dorsal swim) for generating momentum (Cohen et al., 2012). The study by Keys and Lyttle (2007) also demonstrated that it is beneficial to use the underwater flutter kick over the large/slow kick or the small/fast kick using the CFD method.

In all techniques, but mostly in front crawl, swimmers should focus on maximizing their leg extension (it can be called the whiplash effect), as this generates most of the impulse (Cohen et al., 2012). Additionally, they should focus on decreasing D_a_, even knowing that these values change according to important multivariable and that they derive from the variation of swimming along the swimming path. For example, the full dolphin kick strikes a balance between minimizing drag and maximizing thrust while minimizing the physical effort required of the swimmer (Cohen et al., 2012). The periods before course reversals correspond to the maximum drag forces in the direction of the current, so swimmers should be aware of rapid reversals of direction (turns). After starting and turning, increasing stroke frequency (SF) automatically results in a linear increase in speed. All these recommendations described can be useful to optimize the swimmer’s stroke technique (Cohen et al., 2012). It can be assumed that studies with numerical methods have a higher percentage of studies variability (83.3% focus on the front crawl technique), despite a low number of articles that consider D_a_. Most studies that focus on front crawl try to evaluate multivariable (strokes, kicks, and stroke frequency), but always at constant speed (submaximal). In a way, studying the drag while considering these variables has become crucial in these studies. In butterfly, studies focused on the analysis of underwater dolphin kicks concluded that cases of higher kick frequency produced higher peaks of both thrust and drag, as already mentioned in a study by the same author that focuses on the front crawl technique. The extension kick proved to be more important for generating momentum than the flexion kick. The only study that showed a greater range of study was the one by Cohen et al., 2014 in which they compared all swimming techniques for a constant submaximal speed. The authors confirmed that the variation in buoyancy torque is much greater during breaststroke and butterfly than during front crawl and backstroke, having a peak of D_a_ in this phase compared to the other techniques.

It should be noted that numerical methods that measure D_a_ have some limitations. A main limitation of the laser body scanned method is that the volume enveloped by the triangular surface mesh is assumed to be of uniform density on the entire swimmer’s internal volume, which requires a very detailed reproduction of the swimmer’s body, as well as specific kinematics to be accurate (Cohen et al., 2012; Cohen et al., 2015). Another limitation is the approximation of the free surface as a horizontal plane (Cohen et al., 2012; Cohen et al., 2015). Swimming involves rapid accelerations and decelerations of the limbs and the estimates obtained are highly limited (Cohen et al., 2015). Regarding the numerical methods, the body position was limited to a single angle to prevent the swimmer’s model from deviating from its course, which ended up conditioning the trajectory, and the results obtained (Cohen et al., 2020). These limitations constitute a solid basis to be considered in future studies (Cohen et al., 2015; Yuan et al., 2019).

### Experimental methods – D_a_

#### Effects of D_a_ on elite/adult swimmers

Historically, D_a_ was first measured in adult swimmers (e.g., di Prampero et al., 1974; Kolmogorov et al., 1997). Overall, studies noted that drag and C_d_ are about 1.5–2 times greater in D_a_ than in passive conditions (di Prampero et al., 1974; Kolmogorov and Duplishcheva, 1992). In addition, such studies confirmed that better swimming technique reduced D_a_ essentially due to reduced C_d_. Indeed, Kolmogorov et al. (1997) supported the idea that elite swimmers have a greater ability to reduce D_a_ than non-elite swimmers. More recently, Neiva et al. (2021) analyzed the effects of a swimming training mesocycle on the performance and D_a_ of master swimmers in front crawl. The authors concluded that there is an improvement in the performance of master swimmers after 4 weeks of aerobic training. This also resulted in the reduction of D_a_ while swimming mainly at submaximal speeds. Therefore, based on the literature, it can be stated that technical training plays a key role on reducing D_a_. Nonetheless, adult/elite swimmers tend to present greater D_a_ and power needed to overcome drag, especially when the competitive level increases (Zamparo et al., 1996; Takagi et al., 1999; Toussaint et al., 2004; Zamparo et al., 2009; Seifert et al., 2010; Morais et al., 2020a; Kolmogorov et al., 2021). Furthermore, it is known that there are several variables that can directly influence the drag of a swimmer, as expressed in Eq. 3. Such variables are also dependent on external variables and in adults become even more important (Kolmogorov et al., 1997; Kjendlie and Stallman, 2008). Adult/elite swimmers tend to have a larger FSA and a fastest swimming speed than other age groups (Gatta et al., 2015; Kolmogorov et al., 2021; González-Ravé et al., 2022). Body position may also affect the hydrodynamic position and, consequently, D_a_. For example, Formosa et al. (2014) aimed to quantify the influence of the breathing action on D_a_ during swimming. This variation is reported to be large when compared to non-breathing, with a 16%–26% difference in drag force during swimming. The simple act of breathing changes D_a,_ so this variable must also be considered. Others aimed to study D_a_ in a completely different way, examining relationships between IdC and D_a_ assuming that at a constant speed, the average drag is equal to the average propulsion, expressing the idea presented in Eq. 1 (Seifert et al., 2015). In front crawl swimmers, changes in inter-arm coordination were linked to changes in resistance forces when swimming at different speeds. A significant and positive linear regression between IdC and D_a_ was observed (Seifert et al., 2015). Overall, adult/elite swimmers present greater values of D_a_, mainly based on the assumption that they generate a greater metabolic power and mechanical power (Gatta et al., 2016; Kolmogorov et al., 2021).

#### Effects of D_a_ on young swimmers

Active drag has been largely studied in young swimmers over the last decade, specifically in front-crawl (Kjendlie et al., 2004; Barbosa et al., 2010b; Marinho et al., 2010a; Morais et al., 2012; Morais et al., 2021). In studies on this topic, the authors noted that the best performers were also those with the highest D_a_ and CD_a_ (Morais et al., 2015; Barbosa et al., 2019). As expressed in Eq. 2, drag variables are highly dependent on swimming velocity and FSA. This indicates that bigger and faster swimmers are more likely to be under more drag (Barbosa et al., 2019; Silva et al., 2019; Morais et al., 2021). For example, top performers in freestyle sprinting events (front-crawl swim) not only had faster swimming velocity and better kinematics and swimming efficiency but also higher D_a_ (Barbosa et al., 2013; Ribeiro et al., 2017; Barbosa et al., 2019). Thus, D_a_ should be analyzed with some caution in young swimmers. That is, not always an increase in D_a_ can be related to a decrease in performance. As young swimmers go through growth and maturation processes, they increase their body features, more specifically their FSA (Morais et al., 2020a; Morais et al., 2021). Therefore, an increase in body features leads to an increase in swimming velocity as well as in D_a_. Indeed, even in detraining periods this phenomenon occurs. It was noted that during an 11-week detraining period, swimmers increased their FSA (as well as other anthropometric features), and their swimming velocity and D_a_ (Morais et al., 2020a). This highlights the importance that anthropometrics have on swimming velocity and D_a_. On the other hand, performing specific training to improve swimming technique may have a positive impact on the swimmers’ D_a_. For instance, Marinho et al. (2010a) aimed to assess the effects of 8-week of training in young swimmers’ D_a_. Although non-significant differences were found over time, the authors observed that later on, D_a_ and CD_a_ decreased in both genders. Authors argued that 8 weeks of specific swimming training were not sufficient to allow significant improvements on swimming technique (Marinho et al., 2010a). A reason for this non-significant effect can be the anthropometric factor, as young swimmers tend to increase their body dimensions. Furthermore, others aimed to understand the effect of D_a_ on swimming performance during an entire competitive season (Morais et al., 2014). It was noted that depending on the season moment and training periodization, the effect of D_a_ on swimming performance changes. At the beginning of the season, when the main aim is to increase energy, D_a_ is the main determinant of performance. Again, as D_a_ is strongly related to swimming velocity, an increase in swimming velocity will lead to an increase in D_a_. This indicates that coaches of young swimmers should be aware that when the goal is to build energy quickly, this can lead to an increase in D_a_ and CD_a_ (variables related to swimming technique).

#### Sex effect

Studies have compared D_a_ between genders, whether among adults (Pendergast et al., 1977; Kolmogorov et al., 2021) or young swimmers (Barbosa et al., 2013; Barbosa et al., 2015a). In adults, D_a_ and the hydrodynamic coefficient at maximum speed in front crawl showed significant differences between genders (Xin-Feng et al., 2007; Kjendlie and Stallman, 2008; Marinho et al., 2010b). Based on the literature, it can be stated that front crawl is the most analyzed stroke and boys/men are more studied than girls/women. In any case, studies corroborate the idea that the values presented by men compared to women are always higher, in regard to strength and D_a_ (Kolmogorov and Duplishcheva, 1992; Kolmogorov et al., 1997). Initially, Toussaint et al. (1988), who analyzed D_a_ in relation to speed in male and female swimmers, observed that differences in drag force and C_d_ are significant regardless of the speed in question. In addition these differences are also strongly present when all techniques other than front crawl are evaluated, ranging from 48.57 N to 105.88 N in men and 36.25 N–77.01 N (Xin-Feng et al., 2007). In another recent study, in all swimming techniques regarding metabolic power, men showed higher values of metabolic power and greater mechanical efficiency than women (P_ai_ = 3346–3560 W and *e*_*g*_ = 0.062–0.068 vs. *P*_*ai*_ = 2248–2575 W and *e*_*g*_ = 0.049–0.052, correspondingly in this order) (Kolmogorov et al., 2021). In all swimming techniques and for both sexes, values of metabolic power and mechanical power increased with exercise intensity (Pendergast et al., 1977; Kolmogorov et al., 2021). The opposite effect can be observed when technical components are analyzed, namely the influence of breathing on the effect of D_a_ during swimming. Formosa et al. (2014) demonstrated that male participants who exhibited a breathing action caused a greater net drag force (26%) compared to females (16%). This confirms once again that these authors agree with others who state that the increase in D_a_ is not synonymous with worse performance, but simply a natural increase in D_a_ when the performance is also better (Seifert et al., 2010).

In an approach aimed at young swimmers, in relation to all swimming techniques but mostly front crawl, studies show that several anthropometric, kinematic and efficiency variables were significantly higher in boys than in girls (Morais et al., 2012; Barbosa et al., 2013; Barbosa et al., 2015a). Comparing both sexes, Barbosa et al., 2015a indicated that most of the studied variables showed non-significant differences (controlled for sprint performance). Nonetheless, boys performed better than girls due to their larger constitution and natural physical development at these ages (Barbosa et al., 2015a; Barbosa et al., 2015b). Thus, it is evident that adults present much more solid results regarding the comparison between genders because young swimmers are in the process of maturation and growth. These changes in the morphology of young swimmers can constantly affect their hydrodynamics (Morais et al., 2015; Morais et al., 2020a). Likewise, Barbosa et al. (2015b) when analyzing the changes in the hydrodynamic profile of young swimmers throughout a season, realized that no variable had a significant sex effect, due to the fact that throughout the season the hydrodynamic changes occurred in a non-existent linear way. This is clear when analyzing the differences between the beginning and the end of the epoch, as the drag decreased when comparing these moments (−4.37 ± 39.36%). Additionally, the study by Morais et al. (2014) corroborates this statement, confirming that the latent growth curve shows high variability in performance growth and that there is a significant effect on performance growth between genders.

#### Determinants of D_a_

As shown in Eq. 2, D_a_ is dependent of speed, FSA, and C_d_ (in water density, which is constant). Initially di Prampero et al. (1974), pioneered the study of body drag and mechanical efficiency during swimming at speeds of 0.55 and 0.9 m/s. It was shown that the basic approach and the quantitative analysis of swimming proficiency were promising for the study of different forms of locomotion on and under the water surface. The studies by Zamparo et al. (1996) and Clarys (1985) found out that drag in the prone position under the water surface was greater than on the water surface, but the D_a_ reached twice the values of drag in relation to passive drag during swimming. The actual strategy implemented by swimmers to neutralize underwater torque tolerates a large increase in D_a_ (Zamparo et al., 1996; Zamparo et al., 2009). Lyttle et al. (1999) and Lyttle et al. (2000) aimed to analyze the variability and amount of drag at different speeds and depths. They showed that for speeds between 1.6 and 3.1 m/s there were no significant differences in drag forces recorded between the speeds indicated in front crawl, although the coefficient of the measures of variation for these tests indicated high reliability. However, although the differences are not significant, there is a tendency for the drag force to present a difference between the speeds, and it is evident that this force constantly increases (Lyttle et al., 1999; Lyttle et al., 2000). This may be because the applications of the towing device for swimming trawl research are widespread (Lyttle et al., 1999). It is necessary to take specific variables such as establishing the improved speed to start the underwater movement (Lyttle et al., 2000), in which results show that experienced swimmers should glide after pushing the wall until they decelerate to speeds between 2.2 and 1.9 m/s for maximum D_a_ reduction benefits at higher glide speeds.

When comparing the drag/velocity relationship, it was shown that greater drag forces promoted a greater intracycle variation of horizontal velocity (di Prampero et al., 1974; Lyttle et al., 1999). However, as drag depends on the square of velocity, a comparison between swimmers is only relevant when: *1*) it is done at the same absolute velocity, or *2*) the effect of velocity is somehow controlled later (Barbosa et al., 2014). The same authors revealed that there were positive and moderate to strong associations between D_a_ and velocity (intracycle variation) when controlling for the effect of swimming velocity alone in each test (i.e., slip decay velocity method and perturbation velocity method) and swimming speeds in young swimmers as well. Thus, empirical research confirms the theoretical relationship defined for the intracycle variation of horizontal velocity and drag. It can be mentioned that this topic was first argued in the study by di Prampero et al. (1974). The authors reinforced the idea that a change in velocity affects mechanical efficiency because a change in velocity leads to a change in the body’s reaction to water and similar variations in the mechanical efficiency and strength of the body. Another study indicated one relevant technique to estimate D_a_ (Shimonagata et al., 1999). The aim was to clearly show the relationship between swimming speed and D_a_ in front crawl swimming. This study was innovative at the time because the subjects were towed with the D_a_ system (a towing device like the ATM and VPM) in a hydrodynamic position and the subjects swam several attempts at maximum speed (with additional resistance and with towing by the D_a_ system) (Shimonagata et al., 1999). The propulsion, D_a_, and swimming speed present a significant correlation, showing that swimming performance depends both on propulsion and D_a_. Thus, it was essential to verify the existence of a balance between the power generated by the thrust forces and the power needed to overcome the drag forces in front crawl, evaluating the thrust and estimating D_a_ at maximum speed (Gatta et al., 2016). The authors noted that the swimmer’s buoyancy force is very close to the force needed to reduce D_a_ (Gatta et al., 2016; Gatta et al., 2018). Furthermore, another study by Gatta et al. (2018) explored the relationships between mechanical power, thrust power, propulsion efficiency and sprint performance in elite swimmers, reporting that maximum speed in sprint swimming depends on the interaction between power in dry conditions (using a full-body swimming ergometer) and propulsion efficiency. Furthermore, the relationship between maximum velocity and power data was observed with the first method used (in the pool by measuring full tethered swimming force and maximum swimming velocity). The propulsion efficiency is about 40% and the drag is about 1.5 times greater than the values generally reported during passive drag measurements (Gatta et al., 2018). Furthermore, studies such as the one by Shimonagata et al. (1999) showed that swimming speed progresses with increasing propulsion and decreasing D_a_ (Seifert et al., 2010; Gatta et al., 2016; Gatta et al., 2018).

Frontal surface area is another major determinant of D_a_. Knowing that FSA can dynamically change (i.e., variation) during the swimming stroke, researchers set out to assess whether a single FSA measure is adequate to obtain estimates of D_a_ and mechanical power (Morais et al., 2020b; González-Ravé et al., 2022). The authors noted that, in addition to FSA, swimming speed also changes during arm pull in front crawl, in young swimmers of both sexes (Morais et al., 2020b). There was a significant effect on the variation of the two variables of mechanical power and total input power, as well as on the measure of D_a_ (Morais et al., 2020b). Thus, it is worth mentioning that the variation of the FSA throughout the course cycle must be considered in the assessment of D_a_ (Gatta et al., 2015; Morais et al., 2020b; González-Ravé et al., 2022). Furthermore, Kolmogorov et al. (2021) recently determined that the FSA as a component of D_a_ force is the main reason for the differences in maximum speed among the swimming techniques, as there were no relevant differences for the mechanical and propulsion efficiencies. The body position and swimming coordination parameters have an important influence on performance in different swimming strokes (Zamparo et al., 2009; Stosic et al., 2021). In addition, the body position and coordination between the limbs of competitive swimmers during the transition from underwater to surface swimming represented important factors in swimming speed, explaining 15–30% of the variation during the first stroke cycle (Stosic et al., 2021). This reinforces the idea that swimmers must carefully control the inclination and depth of the body and its coordination between the limbs, especially in the first stroke cycle after swimming underwater. Another study showed that waist indentation and buttock curvature can result in greater drag force and influence swimming performance. When differences in C_d_ exist, it may be due to the assumption used in D_a_ methodologies that a swimmer’s velocity remains constant throughout the stroke cycle, rather than fluctuating, particularly in front crawl (Papic et al., 2020). D_a_ and C_d_ had a negative effect on performance, being related to the increase in speed during the act of swimming (Morais et al., 2021). There are also significant correlations between anthropometric variables and D_a_ (Barbosa et al., 2019). In addition, this also happens in front crawl, which results in 69% of the performance in young swimmers, for kinematic variables (efficiency), power in the water and strength on dry land (Morais et al., 2016). After a 10-week break, young swimmers show biomechanical improvements that are mainly explained by their normal growth. SF, D_a,_ and CD_a_ remained unchanged, however, improving performance while maintaining D_a_ is a success factor (Moreira et al., 2014). An earlier study by Sharp and Costill (1989) found that the removal of body hair when swimming in breaststroke reduces the D_a_, and, thus, the physiology cost of swimming. which directly influences the biomechanical performance of swimming.

Checking the external determinants that directly influence the performance and D_a_ of swimmers, Benjanuvatra et al. (2002) concluded that D_a_ values are lower in swimmers who wear competitive suits (Fastskin ^™^) when compared to traditional swimwear (*p* < 0.01), not adopting a specific swimming technique, but a prone position. This variation occurred between 4.8% and 10.2%, and when the underwater flutter kick condition was excluded, all these differences were significant (*p* < 0.05). Moriyama et al. (2021) showed that Jammer-type race swimsuits improve sprint performance to accompany the increase in maximum swimming speed compared to the conventional training swimsuit, in front crawl. In a relatively recent and innovative study, researchers showed that the AquaTrainer^®^ snorkel does not lead to an increase in D_a_ during the front crawl performed over a wide range of speeds (Ribeiro et al., 2016). In addition, other studies have highlighted the importance of analyzing D_a_ as an important variable to be considered in training (Supplementary Table S1), since the most advantageous pulling distance between members of the same team is between 0 and 50 cm from the lead swimmer, where drag is reduced by 21% and 20%, and in which 6% and 7% represent 50 and 100 cm from the lead swimmer. This is true for front crawl, in which maximal and submaximal speeds were analyzed (Kjendlie et al., 2004; Kjendlie and Stallman, 2008; Barbosa et al., 2013).

Drafting is certainly an underdeveloped subject in the literature, but it is known that the effect of distance between swimmers directly influences metabolic and hydrodynamic responses (Chatard and Wilson, 2003). A 4% body difference in underwater volume (*p* < 0.001) between the two techniques in the 3D motion analysis also confirmed that the pressure drag and the friction drag were higher between the techniques (Gonjo et al., 2020). In a pioneering study by Yuan et al. (2019), it was shown that the hydrodynamic interaction between human swimmers can best be described and explained in terms of the interference effect of the wave on the surface of free water.

### Overview and practical applications

It is important to mention that all experimental methods that exist to measure and evaluate D_a_ indicate that there is no agreement among each other regarding the values presented (Toussaint et al., 2004; Formosa et al., 2012). Nonetheless, all authors stated that all equipment measure the same phenomenon, and it can be said that none is more effective than the other (i.e., no gold-standard exists). They simply measure the effects differently and give different results. Some of the methods used were not completely reliable, as there is some margin of error; however, they highlight some issues that coaches should keep in mind not to apply in training or even to apply in an improved way, putting into practice some of the positive points applied in these studies, even if they present some margin of error. For example, the error in the Kolmogorov method can be attributed to the theoretical basis of the equal power assumption (Toussaint et al., 1988; Strojnik et al., 1999). Another analysis corroborated this by showing that the methods used measured essentially the same phenomenon of D_a_ (Toussaint et al., 1988; Toussaint et al., 2004; Formosa et al., 2012). It is probably more appropriate to state that these methods coincidentally underestimate the D_a_ coefficient by a similar magnitude.

D_a_ is defined by the change in characteristics resulting from the flow around different parts of the body following the movement performed. That is why it is essential to have a strategic notion of body movements throughout the stroke cycles, performing in continuous, active and less passive movements. This confirms the need of D_a_ to be further studied and transmitted to coaches. It is also necessary to understand the implications of D_a_ on performance in a homogeneous way. However, it is believed that decomposing total drag into pressure drag, friction drag and wave drag is useful to understand the physical mechanisms that determine drag.

The study of drag is increasingly essential to collaborate with coaches in the process of understanding the fundamental patterns of movement biomechanics to achieve the best performance in swimming (Pendergast et al., 1977). Thus, through the D_a_ research, it was possible to perceive that most studies present very important aspects of swimming technique, more practical movements and easy-to-maneuver variables, such as the distance between swimmers in a training session (aspiration cone), which can be changed depending on the group and type of work considered (Kjendlie et al., 2004; Kjendlie and Stallman, 2008; Barbosa et al., 2013). Furthermore, it will be essential to understand the drag variables regarding each of the four swimming techniques (Xin-Feng et al., 2007; Kjendlie and Stallman, 2008; Marinho et al., 2010b), observing that the values of drag and drag coefficient change completely (highlighting their oscillation and main difference).

Morais et al. (2011) showed that swimming performance in young swimmers is influenced by their swimming efficiency. Therefore, coaches and practitioners of young swimmers should design training programs with a focus on improving technical training (i.e., improving swimming efficiency), indicating that there are data showing that swimming performance is dependent on the SI (an efficiency estimator) and this, in turn, on dv, SL, AS, and D_a_ (Morais et al., 2011). Considering the performance, latent modeling (modeling a latent growth curve) is a comprehensive way of collecting information about the performance of young swimmers over time. The performance improvement was influenced by the different variables, as well as showing an intra and inter subject variability between genders (Morais et al., 2014; Morais et al., 2015). Otherwise, cluster stability is a feasible, comprehensive and informative method of obtaining information about changes in young swimmers over time. Swimmers can be classified into different clusters based on their performance and determinant factors (Morais et al., 2015).

Finally, it can be confirmed that the resistive or drag images found during swimming greatly influence the swimming performance of swimmers of different age groups, including those in elite competition. The benefits of understanding the factors that affect drag are found to improve performance in this sport in different ways that can be analyzed (Sacilotto et al., 2014). However, current techniques used to measure or experimentally estimate drag values are questioned as to their consistency, thus limiting investigations to certain factors. A recent problem is to understand the best method to be applied to study and analyze the variables considered and to determine a context and purpose. Knowing that the range of methodology is wide but not specific, it can bring some confusion to the process, despite being multifaceted (Sacilotto et al., 2014).

## Conclusion

Regarding numerical studies, considering all swimming strokes for a constant submaximal and maximum speed, it was found that the variation in buoyancy torque is much greater during breaststroke and butterfly than during front crawl and backstroke. Experimental studies observed that D_a_ is greater in adults than in children. It is also meaningfully different between sexes with greater values achieved by males. Furthermore, it is evident that speed and FSA are the biggest contributors to the increase in D_a_ (adults have a higher D_a_ value because males and adults tend to have higher speed and FSA). Finally, the technical training dedicated for this purpose makes it possible to reduce D_a_ and CD_a_ and thus improving performance. Through longitudinal studies with pre and post-test it is possible to understand the variability of drag throughout the season and to understand the progression and changes in performance. The intensity of the drag force depends on some factors, among which it is possible to highlight the swimming technique and the morphological characteristics of the subject. The FSA appears as the main morphological characteristic of the subject, having a preponderant role in the determination of the drag force intensity.

It is necessary to understand how the resistive forces in swimming are measured and calculated, because like any method they demonstrate strengths and weaknesses in the evaluation of the techniques described in swimming. Furthermore, it can be indicated that D_a_ is higher in men than in women, while CD_a_ is not clear in the literature as to its significance between genders. Nevertheless, it is known that the CD_a_ between the sexes cannot behave in a different way, because swimming efficiency depends on the drag coefficient. In this sense, the drag coefficient will also show a significant result. Notwithstanding, it should be mentioned that these results and outputs are based on discrete variables measured during an entire trial. Future studies should be conducted to understand how D_a_ and CD_a_ can change within a stroke cycle in all four swimming strokes.

## Data Availability

The original contributions presented in the study are included in the article/Supplementary Material, further inquiries can be directed to the corresponding author.

## Glossary

**ρ**: density of water
**a**: acceleration
**AIS**: assisted towing method
**AP**: pitch angle
**AS**: arm span
**ATM**: assistant towing method
**C_d_**: coefficient of drag
**CD_a_**: coefficient of active drag
**CFD**: computational fluid dynamics
**CoV**: coefficient of variation
**C_x_**: hydrodynamic coefficient
**D**: drag force
**D_a_**: active drag
**D_p_**: passive drag
**dv**: speed fluctuation
**EM**: experimental method
**F**: total drag force
**F_d_**: active drag force
**F_f_**: friction component
F_p_: pressure component
F_r_: froude number
**FSA**: frontal surface area
**F_w_**: wave component
**ICC**: intra-class correlation coefficients
**IdC**: coordination index
**IVV**: intra-cyclic velocity variations
**K**: Cohen’s Kappa
**M**: total mass
**MAD**: measuring D_a_ system
**MRT**: residual thrust measured values
**NM**: numerical method
**P_ai_**: metabolic power (power input)
**P_k_**: mechanical power to transfer
**S**: projected frontal surface area
**SB**: sweep-back angle
**SE**: stroke efficiency
**SF**: stroke frequency
**SI**: stroke index
**SL**: stroke length
**SPH**: Coupled biomechanical-smoothed particle hydrodynamics
**T**: total propulsive force
**TDI**: technique drag index
**TTSA**: trunk transverse surface
**v**: swimming speed
**VO_2max_**: maximal oxygen uptake
**VPM**: speed perturbation method
**WS**: whole stroke
