# Supplementary material for: Numerical and experimental methods used to evaluate active drag in swimming: A systematic narrative review
Source: Front Physiol. 2022 Oct 20;13:938658. doi: 10.3389/fphys.2022.938658 (PMC9630912; doi:10.3389/fphys.2022.938658)
Supplement: Supplementary file 1 [file Table1.docx]

Supplementary Table 1. Summary of the objective, sample demographics, and main results of the studies related with D_a_ for experimental methods.

| Study (year) | Objective | Subjects (age and competitive level) | Results | Delphi score  Mean±1SD |
| --- | --- | --- | --- | --- |
| Barbosa et al., (2010b) | Develop a structural equation modeling (ie, path analysis) for D_a_ force based on selected anthropometric, hydrodynamic, and biomechanical variables in competitive young swimmers | 16 male swimmers (12.50±0.51 years)  with several competitive levels | Studying the D_a_, it is advisable to increase the fit of the model and develop new equations specific for young swimmers, instead of using the models with adult/elite swimmers. 95% of the D_a_ was explained by the variables used in the model | 6.0±0.0 |
| Barbosa et al., (2013) | Compare speed fluctuation and drag force in young swimmers | 12 boys (14.42±1.24 years)  11 girls (12.73±0.79 years)  Voluntary swimmers at regional and national level | For D_a_ a the boys showed significantly higher values in the fluctuation (Δ=35.77; Z=−2.400; p=0.02; d=1.34). There are no differences between the sexes for C_da_ (Δ=1.49; Z=−0.739; p=0.49; d=0.04). When controlling for velocity effects, there are moderate-strong and positive associations between velocity fluctuation and Da (the greater the velocity fluctuation, the greater the drag forces) | 6.0±0.0 |
| Barbosa et al., (2014) | Classify swimmers based on kinematics, hydrodynamics, and anthropometrics | 77 young swimmers (34 girls and 33 boys, 12.83±1.26 years and 1–2 Tanner stages) with at least 4 years of experience in competitive swimming | FSA was measured with photogrammetric technique on land and in hydrodynamic position (C_d_). Kinematics, hydrodynamics and anthropometrics are determinant domains in which to classify and characterize young swimmers’ profiles). C_da_ was associated with high hydrodynamic profile (F=21.025; p<0.001) | 6.0±0.0 |
| Barbosa et al., (2015a) | Compare swimming power output between boys and girls and to model the relationship between swimming power output and sprinting performance in young swimmers | 100 young swimmers (49 boys and 51 girls aged 12.51±0.77 years and 12.24±0.71 years, respectively; all in Tanner stages 1–2 by self-report). The sample included national record holders, national champions, and other talent swimmers | There was a significant and strong relationship between sprinting performance and power, as well as D_a_ (0.02≤ p ≤0.04; 0.37≤ η^2^ ≤0.48). Boys have greater power and thus perform better than girls. There were no sex differences for C_da_ | 6.0±0.0 |
| Barbosa et al., (2015b) | Analyze the changes in the hydrodynamic profile of young swimmers over a competitive season and to compare the variations according to a well-designed training periodization | 25 talented swimmers (13 boys: 12.64±0.81 years and 68.02±5.49 s personal best in short-course swimming pool at front crawl; 12 girls: 12.43±0.78 years old and 71.23±5.45 s personal best; both sexes in Tanner stages 1–2 by self-report at the beginning of the research) | No variable presented a significant sex effect. Swimming efficiency improved between moment 1 and 2. There was a trend for both D_a_ increase from moment 1 and 2, but being lower at moment 3 than at moment 1 (−4.37±39.36%). Over the course of a season, hydrodynamic changes occur in a non-linear fashion | 6.0±0.0 |
| Barbosa et al., (2019) | Compare the anthropometrics, biomechanics and energetics in young swimmers of different competitive levels | 75 boys (between 11 and 13 years) with a broad range of performances were ranked based on their personal best time in the men's 100m front crawl event and then split-up into three tiers (Tier-1, i.e., top-tier, best performers; Tier-2, mid-tier; Tier-3, lower-tier) | Level 1 swimmers have higher drag than their peers due to their faster swimming. As a result, C_d_ is smaller, and there were significant variations for most of the selected variables (particularly for D_a_), with a moderate-large effect size (D_a_= (T1vT2 = p<0,001 (1.13 N); T1vT3 = p<0,001 (0.99 N)) | 6.0±0.0 |
| Benjanuvatra et al., (2002) | Cross-sectional comparison between D_a_ and D_p_ buoyancy | 9 (5 boys and 4 girls) national level swimmers | Comparing the Fastskin TM suits and the normal bathing suits, there were lower D_a_ force values (10.2%). There was also a significant difference for surface towing in the three speeds of 1.6, 2.2 and 2.8 m/s | 6.0±0.0 |
| Chatard & Wilson (2003) | Investigate the effect of the distance separating the lead and draft swimmers on the metabolic and hydrodynamic responses of the draft swimmer | Part I - 11 males (24±5 years)  Part II - 6 males (23±4 years)  Competitive swimmers and triathletes | For drag, the most advantageous draft distances were 0 and 50 cm behind the lead swimmer's toes, reducing drag by 21% and 20%, respectively. In the lateral design drag was significantly reduced by 6% and 7%, respectively, at 50 and 100 cm from the lead swimmer's hands | 6.0±0.0 |
| Clarys (1985) | In a quadruple approach we have was suggested the ergonomics links between fundamental hydrodynamics, applied dynamics of swimming, electromyographical aspects and specific training | Fundamental and applied hydrodynamics were investigated in a Dutch Marine Ship model test station (in one person) | It was found that drag in a prone position under the water surface was greater than at the water surface, but D_a_ while swimming reached twice the drag values of any D_p_ condition. This indicates that body form has no influence on drag and propulsion | 5.5±0.7 |
| di Prampero et al., (1974) | The body drag of a swimmer was determined by adding (or subtracting) extra drag loads to (or from) swimmers moving at known speeds | The experiments were performed on 10 well-trained male college students swimming the overarm crawl at 0.55 and 0.90 m/s | The results indicate that both drag (D_a_) and mechanical efficiency (e) are on the average 30 % higher than previous estimates based on the drag of passively towed subjects. The ratio, e/D_a_, which is shown to be equal to the ratio of v/VO_2_, provides a valuable tool in estimating swimming performance: it amounts on the average to 0.8 kg^-1^×10^-2^ corresponding to 58.5 ml O_2_.rn^-1^), independent of the speed over the range studied | 5.0±00 |
| Formosa et al., (2012) | Compare measuring D_a_ (MAD) and ATM | 9 intermediate-level swimmers (19.7±4.4 years) completed front crawl trials with both systems during one session | The mean Da was 82.3 N (74.0-90.6 N) for the MAD (measured at various speeds) system and 148.3 N (127.5-169.1 N) for the ATM system. These differences were attributed to variations in swimming style within each measurement system | 5.5±0.7 |
| Formosa et al., (2014) | Quantify the influence of the breathing action on net drag forces during front crawl swimming | 20 elite (n=10 mal; 21.3±3.06 years; FINA point front crawl 900±69; n=10 female; 21.3±3.13 years, FINA point front crawl 917±69) national swimming team | Male demonstrated a 26 % (p<0.001, d=−1.01) in mean net drag forces between conditions. There were statistical and large differences (p<0.001, d=−1.60) in minimum net drag force between the breathing stroke side within the breathing and non-breathing conditions. Female produced a 16 % diff erence in overall net drag force between the testing conditions (p=0.01, d=−0.55). Minimum (p=0.08, d=−0.41) (p=0.16, d=0.33) and maximal (p=0.15, d=−0.43) net drag forces were not statistically different | 6.0±00 |
| Gatta et al., (2015) | Use the planimetric method to determine frontal area throughout the stroke cycle in the four swimming strokes as well as during "streamlined leg kicking" | 10 highly trained swimmers, 6 male (21.3±0.5 years) and 4 female (20.8±0.5), competing at national level | D_a_ was estimated based on the average AP values, as calculated for a full cycle in each condition. D_a_ is the lowest in the "streamlined leg kicking" condition (D_a_ =19.5v^2^), is similar in front crawl (D_a_=30.0 v^2^), backstroke (D_a_=26.9 v^2^) and butterfly (D_a_=28.5 v^2^) and is the largest in the breaststroke (D_a_=37.5 v^2^) | 6.0±00 |
| Gatta et al., (2016) | Verify whether there is a balance between the power generated by thrust forces and the power needed to overcome drag forces in front crawl swimming (using a tethered test) assessing thrust force and estimating D_a_ based on measures of D_p_ (at maximal speed) | 10 front crawl high-level male swimmers (23.5±3.4 years of age); long-course 50 m and 100 m front crawl personal best times are 22.5±0.6 s and 49.5±1.4 s, respectively (representing 93±2 and 95±3% of the World Record) | No significant differences were observed between tethered swimming (Pt = 399±56 W) and D_a_ strength (Pd = 400±57 W), but with a strong correlation (R = 0.95, p<0.001). Showing that the swimmer's thrust force is close to the force needed to reduce D_a_ | 6.0±00 |
| Gatta et al., (2018) | Was to explore the relationships between mechanical power, thrust power, propelling efficiency and sprint performance in elite swimmers | 12 elite male swimmers (22.8±3.5 years); they were part of the National Italian Team and were competing in short-distance sprint events. The long-course 50 m and 100 m freestyle personal best times are 22.5±0.6 and 49.5±1.3s | (1) by using a whole-body swimming ergometer (W'TOT) and (2) in by measuring full tethered swimming force and maximal swimming velocity. Speed-specific drag, was found to be 36.2±3.3 N. Support the capability of a whole-body ergometer to estimate W'TOT as well as the finding that propelling efficiency should be about 40% in these experimental conditions. | 6.0±00 |
| Gonjo et al., (2020) | Investigate differences in Fr efficiency and D_a_ between front crawl and backstroke at the same speed (Through 3D motion analysis) | 10 male competitive swimmers (17.47±1.00 years), and their best records were 54.50±1.23 and 60.56±1.29 s in 100 m front crawl and backstroke, respectively | Swimmers had 8.3% longer SL, 5.4% lower SF, 14.3% smaller IdC, and 30.8% higher ηF in front crawl than backstroke in the 3D motion analysis (all p<0.01). Backstroke had 25% larger D_a_ at 1.2 m/s than front crawl (p<0.01) in the MRT trial | 6.0±00 |
| González-Ravé et al., (2022) | Determine the FSA of swimmers by means of an automated vision system. | 1 regional male swimmer (age: 20 years; body mass 68 kg; height: 173 cm; training hours: 9 h per week). The swimmer was specialized in individual medley events | The resistive forces that influence the swimmer in the water include form, wave drag and frictional drag, which are influenced by the swimmer’s velocity, boundary layer, shape, size, and the FSA | 6.0±00 |
| Hazrati et al., (2016) | Determine the reliability of the ATM method approach using a fluctuating speed tow | 12 elite swimmers (5 males and 7 females, 17.7±2.9 years) | The reliability was determined using within-participant ICC within each day and between the days. This study identified that the ATM method with fluctuating speed had moderate reliability within-participant trials on values in a single day but high reliability for the average D_a_ values across different days (ICC=0.93). Identified that the ATM method with fluctuating speed had moderate reliability trials on values in a single day but high reliability for the average D_a_ values across different days | 6.0±00 |
| Hazrati et al., (2018) | Know how much uncertainty in the D_a_ value may be produced by each component variable using the ATM with the fluctuating velocity | 12 national and state level swimmers (5 males and 7 females, 17.7±2.9 years) were recruited for this study. | The result of the uncertainty of the velocity exponent (1.8–2.6) indicated a contribution of about 6% error in D_a_. The contribution of unequal power output showed that if a power changed 7.5% between conditions, it would lead to about 30% error in calculated drag | 6.0±00 |
| Hollander et al., (1986) | A system has been designed, built and tested in order to measure D_a_ during front crawl swimming | The male subject, who was an Olympic Games participant at Los Angeles in 1984, performed five tests on five different days | At a mean speed of 1.55 m/s the mean force was 66.3 N. The accuracy of this force measured on one subject at different days was 4.1 N. At constant speed, the mean propulsive force is equal to the opposing, D_a_ force | 5.0±00 |
| Kjendile et al., (2004) | Examine the influence of several explanatory factors: anthropometry, buoyancy, passive underwater torque, drag and swimming technique on the energy cost of swimming front crawl in children and adults | 10 children 11.7±0.8 years  13 adults 21.4±3.7 years | Anthropometric features and active drag were responsible for the energy cost in children and adults. When normalized for body size, non-significant differences were found. Active drag at maximal speed was higher in adults [106 (68) N at a speed of 1.79 (0.05) m/s] than children [29 (9) N for 1.41 (0 ,10) m/s, p<0.001] | 5.0±00 |
| Kjendile & Stallman, (2008) | Compare drag in swimming children and adults, quantify technique using the TDI, and use the F_r_ to study whether children or adults reach hull speed at maximal velocity (F_r_) | 9 children 11.7±0.8 years  13 adults 21.4±3.7 years | The children (CD_a_=0.66±0.14) had significantly lower D_a_ factor compared with the adults (CD_a_=0.84±0.46) Technique drag index could not detect any differences in between the two groups, owing to the adults swimming maximally at a higher F_r_, increasing the wave drag, and masking the effect of better technique | 6.0±00 |
| Kolmogorov & Duplishcheva, (1992) | Compare the time of the same distance swum with and without an added resistance, under the assumption of an equal power output | 73 swimmers (competitive level) | D_a_ in females ranged from 65.51 to 37.79N in backstroke, 93.56 to 45.19 N in breaststroke, 83.04 to 37.78 N in dolphin and 69.78 to 31.16 N in the front-crawl. In Males ranged from 146.28 to 46.36 N in back-stroke, 176.87 to 55.61 N in breaststroke, 156.09 to 46.95 N in dolphin and 167.11 to 42.23 N in front-crawl | 6.0±00 |
| Kolmogorov et al., (1997) | Describe the magnitudes of D_a_, C_d_, and power output in the four swimming strokes for athletes of different performance levels, sexes, and ages at maximal swimming velocities | This research was conducted over a 5 year period leading up to the 1992 Olympic Games in Barcelona. The subjects included 310 females and 487 males ranging in age from 10 to 28 years | In all swimming strokes at the maximal absolute velocity, men experienced greater C_d_ well as much greater power outputs (virtually twice as much) than women. Within each stroke, the most important factor for reducing D_a_ appeared to be individual biomechanical technique | 6.0±00 |
| Kolmogorov et al., (2021) | Determine the main biophysical (energetic and biomechanical) reasons for the observed differences in maximal swimming velocity between the different competitive swimming techniques | 8 elite swimmers (4 females and 4 males), each of whom specialized in different swimming techniques and ranked among the top 10 in the world in the 100 m event in their swimming specialty | At the last stage of the test, in all techniques, men demonstrated higher values of metabolic power (P_ai_=3346–3560 W) and higher mechanical efficiency (e_g_=0.062–0.068) than women (P_ai_=2248–2575 W; e_g_=0.049–0.052). The main reason for the differences in maximal swimming velocity between different techniques is the frontal component of D_a_ force | 6.0±00 |
| Lyttle et al., (1999) | A towing device was designed to quantify drag experienced by swimmers at predetermined velocities and depths | A servo controlled mechanical winch was used to tow the swimmers along the length of a 25m pool and a pulley arrangement allowed the towing forces to be essentially horizontal at the required depth | Initial results indicate that the towing device effectively and reliably calculated the drag forces experienced by swimmers in a prone, streamlined position at velocities between 1.6 and 3.1 m/s. The required depth for towing could also be accurately established. The towing device will enable reliable information on the swimmer's D_a_ to be collected | 5.0±1.4 |
| Lyttle et al., (2000) | Establish an appropriate speed for initiating underwater flutter kicking, as well as the most effective gliding position and kicking technique to be applied after a turn | 16 experienced adult male swimmers (19.3±2.1 years). Swimmer’s experience (5.3±1.8 years) and national competition (3.8±2.0 years) | Demonstrated an optimal range of speeds (1.9 to 2.2 m/s) at which to begin underwater flutter kicking to prevent energy loss from excessive D_a_. There appears to be no significant advantage in using one streamlining technique over another or in using one kicking style over another | 6.0±00 |
| Marinho et al., (2010a) | Assess the effects of 8-weeks of training on D_a_ in young swimmers of both sexes | 8 girls and 12 boys belonging to the same swimming team and the previous two seasons with regular competitive participation | After 8 weeks of training, mean D_a_ (drag force and drag coefficient) decreased in girls and boys (girls: 29.18±15.24 N vs. 27.50±10.36 N, 0.35±0.23 vs. 0.30±0.09; boys: 38.30±17.49 N vs. 36.35±13.12 N, 0.33±0.11 vs. 0.31±0.09; p>0.05). No significant differences were found between the two trials (34.66±16.84 N vs. 32.81±12.60 N, 0.34±0.16 vs. 0.31±0.09). These differences corresponded to a 5.34±0.46 % and 8.82±0.83 % | 6.0±00 |
| Marinho et al., (2010b) | Study was to assess the effects of 8 weeks of training on D_a_ in young female swimmers | 8 females age group swimmers belonging to the same swimming club with good competition level (11.63±0.52 years old) | After 8 weeks of training, mean D_a_ decreased, although no significant differences were found between the two trials (29.18±15.24 N vs. 27.50±10.36 N). No significant differences were observed in swimming velocity between the two trials (1.23±0.13 N vs. 1.25±0.15 N) | 6.0±00 |
| Masset et al., (1999) | Analyze the 3D analysis of the backstroke style and the views were digitally broken down frame by frame in order to obtain smoothed 3D trajectories for the hip and wrist | 32 male swimmers were filmed during the National and International Championships 100m backstroke event using two underwater camcorders | The 3D hand movement allowed the swimmer to create lift and drag forces to propel the body forwards. Small deviations of the body were observed, albeit with significant individual variations, along the lateral and vertical axes in reaction to the wrist action. These unnecessary body displacements may induce an increase in D_a_ | 5.5±0.7 |
| Morais et al., (2011) | Compute and validate TTSA area estimation equations to assess the swimmer’s drag force in both sexes | 264 subjects (152 males (between 10-32 years) and 112 females (between 9-27 years)). All subjects were competitive swimmers with regular participation | No significant differences between assessed and estimated mean TTSA. Coefficients of determination for the linear regression models between assessed and estimated TTSA were R2 = 0.39 for males and R2 = 0.55 for females. More than 80% of the plots were within the 95% interval confidence for the Bland-Altman analysis in both sexes | 6.0±00 |
| Morais et al., (2012) | Develop a structural equation model (i.e., a confirmatory technique that analyzes relationships among observed variables) for young swimmer performance | 114 subjects (73 boys and 41 girls of mean age of 12.31±1.09 years and Tanner stages 1–2) were evaluated | Main data showed that swimming performance is dependent on SI (an efficiency estimator) and this in turn on the dv, SL, AS and D_a_. Swimming efficiency improvement is related to a decrease in the dv and an increase of the SL and AS. The increase in the D_a_ is a result of the increase in swimming velocity. For boys D_a_ ranged between 11.81 N and 73.15 N. For girls, D_a_ varied between 16.49 N and 54.59 N | 6.0±00 |
| Morais et al., (2014) | Model a latent growth curve of young swimmers’ performance and biomechanics over a season | 30 young swimmers (14 boys: 12.33±0.65 years, 284.85±67.48 FINA points at the short-course meter 100 m front crawl; and 16 girls: 11.15±0.55 years, 322.56±45.18 FINA points at the short-course meter 100 m front crawl) | Latent growth curve modeling showed a high inter- and intra-subject variability in the performance growth. Sex had a significant effect at the baseline and during the performance growth. In each evaluation moment, different variables had a meaningful effect on performance (M1: D_a_, β=−0.62; M2: D_a_, β=−0.53; M3: η_p_, β=0.59; M4: SF, β=−0.57; all p<0.001) | 6.0±00 |
| Morais et al., (2015) | Apply a new method to identify, classify, and follow up young swimmers, based on their performance and its determinant factors over a season, and to analyze the swimmers’ stability over a competitive season | 33 young swimmers (11.8±0.7 years; 15 boys (12.3±0.6 y); 18 girls (11.7±0.9 y); Tanner stages 1–2 by self-report) | Cluster 3 was characterized by high C_d_ (M1); SI, ηp, and v (M2); and chest perimeter, AS and average performance. Stepwise discriminant analysis revealed that 100%, 94%, and 85% of original groups were correctly classified for the 1st, 2nd, and 3rd (0.11≤ Λ ≤0.80; 5.64≤ χ2 ≤63.40; 0.001< p ≤0.68). Membership of clusters was moderately stable over the season | 6.0±00 |
| Morais et al., (2016) | Compute a swimming performance confirmatory model based on biomechanical parameters | 100 young swimmers (12.3±0.74 years; 49 boys: 12.5±0.76 years; 51 girls: 12.2±0.71 years; both sexes in Tanner stages 1–2 by self-report) | 69% of the performance of young swimmers is explained through the model (strength on dry land, power in water and kinematic variables). Dry-land strength has a positive and large effect on the in-water power output, and in turn on the stroke mechanics (v and ηp), thus enhancing the performance, where D_a_ is included in the predominant factor | 6.0±00 |
| Morais et al., (2020a) | Analyse the detraining process that occurs during a season break, and its influence on the performance, anthropometrics, and biomechanics of young swimmers | 54 young swimmers (22 boys: 12.79±0.71 years; 32 girls: 11.78±0.85 years; maturation stage: Tanner 1–2) | The CD_a_ increase between M1 and M2 may reflect the swimmers’ need for a higher physiological power to overcome drag at a given swim velocity. Despite the v increase, girls did decrease slightly for the D_a_ (D_a_ t-test=−0.77 (0.447) N). The CD_a_ increased between the moments (boys and girls) | 6.0±00 |
| Morais et al., (2020b) | Compare D_a_ calculation between a single land-based measurement of FSA and in-water FSA measures obtained at key events of the arm pull and compare mechanical power variables computed based on these two approaches | 17 good national level swimmers (11 male; 6 female; 16.15±0.94 years). FSA was measured using the Velocity Perturbation method | Besides the FSA, swim speed also changes during the crawl arm pull, having a significant effect on the D_a_ measure and on the mechanical power and total input power variables. The FSA was higher than when assuming a nonvariation (p<0.001) and D_a_ was also significantly higher than when assuming a nonvariation (p*=*0.002) | 6.0±00 |
| Morais et al., (2021) | Classify, identify and follow-up young swimmers’ performance and its biomechanical determinants during two competitive seasons and analyze the individual variations of each swimmer | 30 young swimmers (14 boys: 12.70±0.63 years; 16 girls: 11.72±0.71 years) | The performance improved between moments of assessment in all clusters (cluster 1 (“talented”), cluster 2 (“proficient”), and cluster 3 (“non-proficient”)). The hydrodynamic variables (D_a_ and CD_a_) increased, but this fact may be related to the increase in swimming velocity, which is directly related to the drag | 6.0±00 |
| Moreira et al., (2014) | Analyse the effect of growth during a summer break on biomechanical profile of talented swimmers | 25 talented swimmers including 12 boys (12.8±0.9 years; 50.09±10.13 kg) and 13 girls (12.0±0.9 years; 49.42±7.47 kg) | The stroke frequency, D_a_, and C_d_ remained unchanged. When controlling the effect of growth, no significant variation was determined on the biomechanical variables. Young talented swimmers present biomechanical improvements after a 10-week break, which are mainly explained by their normal growth, inclusive for D_a_ (Pearson correlation 0.40 (p=0.06) to 0.52 (p=0.08)) and C_da_ (0.39 (p=0.06) to -0.07 (p=0.55)) | 6.0±00 |
| Moriyama et al., (2021) | Investigate the effects of jammer-type racing swimsuits on swimming performance during arm-stroke-only (pull) and whole-body stroke (swim) in 25 m front-crawl with maximal effort | 12 well-trained male collegiate swimmers (21.3±1.4 years, 512.8±83.1 FIFA points) | Jammer-type racing swimsuits would improve sprint performance to accompany with increase in the maximal swimming velocity compared with the conventional training swimsuit. Although it was not possible to identify the factors that improved the swimming velocity, the drag may be an explanatory factor. However, the results do not confirm the swimsuit’s function of supporting the improvement to D_a_ (p>0.124) | 6.0±00 |
| Narita et al., (2017) | Develop a new method for evaluating the drag in front-crawl swimming at various velocities and at full stroke (MRT-method) | 6 male competitive swimmers (20.0±1.0 years and 52.6±0.6 seconds performance time at 100 m front crawl) | D_a_ was estimated in five-stages for velocities ranging from 1.0 to 1.4 m/s. D_a_ (D_a_=32.3 v3.3, N=30, R^2^=0.90) was larger than D_p_ (D_p_=23.5 v2.0, N=42, R^2^=0.89) and the variability in D_a_ for the two swimmers was 6.5% and 3.0% | 6.0±00 |
| Narita et al., (2018a) | Examine the effect of leg kick on the resistance force in front-crawl swimming | 7 male competitive swimmers (20.0±0.9 years and and 115.5±3.0 seconds performance time at 200 m front crawl) | For both the WS and AS at both swimming velocities, CD_a_ was found to be about 1.6–1.9 times larger than in passive conditions. In contrast, although leg movement did not cause a difference in drag coefficient for front-crawl swimming, there was a large effect size (d=1.43) at 1.3 m/s | 6.0±00 |
| Neiva et al., (2021) | Analyze the effects of a swimming training mesocycle in master swimmers’ performance and D_a_ | 22 master swimmers (39.87±6.10 years and 6.47±5.41 years of experience). Male (n=16) and female (n=6) swimmers | Maximal, mean and minimum front crawl speeds improved from pre- to post-training and the speed decrease along the 25 m test lowered after the training period (82.5±76.3%, p=0.01). The training mesocycle caused a reduction in the D_a_ at speeds corresponding to 70% (5.0±3.9%), 80% (5.6±4.0%), and 90% (5.9±4.0%), but not at 100% (5.9±6.7%), of the swimmers’ maximal exertions in the 25 m test | 6.0±00 |
| Papic et al., (2020) | Determine the influence of torso morphology on maximal instantaneous hydrodynamic resistance in front crawl swimming | 15 Scottish national and international level male swimmers (7 sprint specialists (18.3±2.3 years) and 8 distance specialists (17.5±2.5 years)). 10 male national level Portuguese swimmers (17.47±1.00 years) | Indentation at the waist and curvature of the buttocks may result in greater drag force and influence swimming performance. Instantaneous C_d_ were significantly greater than those derived from front crawl D_a_ analysis throughout the literature. Differences in drag coefficients may be due to the assumption used in D_a_ methodologies, that a swimmer’s velocity remains constant throughout the stroke cycle, rather than fluctuating | 6.0±00 |
| Pendergast et al., (1977) | Body drag and mechanical efficiency were measured from the relationship between extra oxygen consumption and extra drag loads | 42 male (16-24 years) and 22 female (17-21 years) competitive swimmers (college level) | Using speeds ranging from 0.4 to 1.2 m/s. Drag increased from 3.4 (1.9) kg at 0.5 m/s to 8.2 (7.0) kg at 1.2 m/s, with Drag of women being significantly less (p<0.05) than that of men. Drag and efficiency of swimming was shown to be identical to the directly measured energy cost of swimming one unit distance, VO_2_/Drag, and was independent of the velocity up to 1.2 m/s. | 5.5±0.7 |
| Peterson Silveira et al., (2019) | Compare different methods to assess the arm SE, identify biophysical adaptations to swimming on the MAD System and the main biophysical predictors of maximal swimming speed in the 200 m front crawl using the arms only | 14 national level competitive swimmers (eight males, six females, age: 17.3±2.2 years) | Both methods to assess on the MAD System differed (p<0.001) from the expected values for this condition (ηF=1), with the speed-based method providing the closest values (ηF~0.96). The main assumption of this method is that the D_a_ and the effective force applied by the hand are the same for a given constant speed. The speed-based method provides confirm that swimming performance depends on the balance of biomechanical and bioenergetic parameters | 6.0±0.0 |
| Poizat et al., (2010) | Evaluate the usability of the Measuring D_a_ (MAD) system, a technical device for biomechanical evaluation and performance analysis | 3 international male swimmers volunteered. Age, experience, and time on 100m front crawl (in 50-m pool) were (21.3±1.2 years, 10.7±2.1 years, 50.9±1.2 seconds) | The results are presented in two stages: (a) the concerns and modalities of using, and (b) use sensations. One of the most important results was that these components changed according to the swimmer's speed when using the MAD system | 5.5±0.7 |
| Ribeiro et al., (2016) | Verify the use of the new AquaTrainer® respiratory snorkel lead to an increase of front crawl hydrodynamic drag and whether the constraint of using an adapted turning technique influences its corresponding turning time | 12 national-level swimmers (age: 22.2±6.3 years, training background: 7.6±5.4 years and training frequency: ≥7 units per week, percentage of the 100m world record: 83.16±16.42%) | Front crawl swimming with snorkel using the open turn implied an increase in turning time of 14.2 and 5.1% than the tumble turn and open turn without the apparatus (p<0.01). AquaTrainer® snorkel does not lead to increased D_a_ during forward tracking (performed at various speeds) and the metabolic energy required to overcome drag will not be affected | 6.0±0.0 |
| Ribeiro et al., (2017) | Examine how high- and low-speed swimmers organize biomechanical, energetic and coordinative factors throughout extreme intensity swim | 16 male swimmers were divided in two performance level groups (21.1±3.3 vs. 18.6±1.6 years of age, 115±4% vs. 124±3% of 100-m front crawl world record time for high- (n=8) and low-speed (n=8) | Performing at extreme intensity led better level swimmers to achieve superior speed due to higher power and propelling efficiency, with consequent ability to swim at higher stroke frequencies. This imposes specific constraints, resulting in a distinct IdC magnitude and profile between groups and in turn a smaller C_d_, although D_a_ is naturally larger | 6.0±0.0 |
| Schreven et al., (2013) | Determine the effect of inter-pad distance on D_a_ at a given speed | 11 competitive swimmers (20.0±6.2) | Variation of 16% in inter-pad distance (14% change in stroke frequency) revealed no significant difference in calculated D_a_ between different inter-pad distances and a low (<5%) average coefficient of variation over different inter-pad distances was found. D_a_ was 23.60 N (95% CI 22.19, 25.02) greater at 1.55 m/s compared to 1.25 m/s | 6.0±0.0 |
| Seifert et al., (2010) | Examine how a combined group of national and regional swimmers organized their stroke (i.e., their inter-limb coordination and their power output application) to increase v, and more particularly how the national swimmers changed their coordination when swimming at higher speeds | 14 French male swimmers (7 national swimmers 21.9±4.2 years, training 20.0±1.0 h per week and had 11.6±2.5 years of practice; 7 regional swimmers 21.7±3.1 years, training 9.4±4.3 h per week and had 8.0±4.3 years of practice) | Both groups increased IdC and hand speed (u) and applied greater Pd to overcome D_a_ (F(7.96)=30.92, p<0.05) with speed increases (p<0.05). The regional swimmers exhibited a higher u and lower SI, IVV, and v2/u2 compared to national swimmers (F(1.96)=5.77, p<0.05 and Pd F(1.96)=7.81, p<0.05), which revealed lower effectiveness to generate propulsion, suggesting that technique is a major determinant of swimming performance | 6.0±0.0 |
| Seifert et al., (2015) | Examine the relationships between the IdC and D_a_ assuming that at constant average speed, average drag equals average propulsion | 20 French national volunteer male front-crawl swimmers (21.6±2.4 years, mean year of practice: 11.4±3.4) | IdC was linked to D_a_ by linear regression (IdC=0.246; R^2^=0.88, p<0.05); swimmers switched from catch-up to superposition coordination mode at a speed of similar to 1.55 m/s where average D_a_ is similar to 110 N. Inter-individual analysis showed that high IdC did not relate to a high propulsive efficiency suggesting an individual optimization of force and power generation is at play to reach high speeds | 6.0±0.0 |
| Sharp & Costill, (1989) | Assess the effect of shaving body hair on physiological responses to free and tethered breaststroke swimming and to examine the effect on velocity decay during a prone underwater | 9 male collegiate swimmers (21.0±1.0) | Removing body hair significantly reduced the rate of velocity decay during a prone glide after a maximal underwater leg push-off. It is concluded that removing body hair reduces D_a_, thereby decreasing the physiological cost of swimming | 5.5±0.7 |
| Silva et al., (2019) | Conduct a multivariate analysis of young swimmers’ sprint performance to determine which are the key variables when analyzing the effect of sex and skill | 23 male (15.7±0.8 years) and 26 female (14.5±0.8 years) swimmers at the national level in the postpubertal maturational stage (Stage 4). | Biomechanical variables play a crucial influencing factor in sprint performance (at these ages), with swimmers focusing on performing better and more efficient technique (through better SI and IVV). Furthermore, sex proved to be an important factor influencing sprint performance. | 6.0±0.0 |
| Silva et al., (2007) | Examine the effects of 21 days of creatine supplementation (CS) on swimming performance on performance related hydrodynamic variables and on body composition in national junior female swimmers, during the last period of training preparation | 16 healthy competitive national level female swimmers (CS group n=8 and placebo supplementation (PL) group n=8, 16.3±1.8 years for CS group and 15.7±1.2 years for PL group) | Significant differences were observed in hydrodynamic values: the CS group showed a significant reduction (≈25%), in D_a_ force or F_d_, C_x_ and power output values, when comparing pretest with post-test. These data suggest that 21 days of CS produced significant effects on gross and/or propelling efficiency during swimming in female athletes. However, CS did not influence performance, body weight and body composition | 6.0±0.0 |
| Silveira et al., (2019) | Compare different methods to assess the arm SE and to identify biophysical adaptations to swimming on the MAD System and the main biophysical predictors of maximal swimming speed in the 200m front crawl using the arms only | 14 national level competitive swimmers (eight males, six females, 17.3±2.2 years) | Both methods to assess SE on the MAD System differed (≈4%, p<0.001). In the free-swimming condition, the power-based, speed, and paddle-wheel efficiencies were significantly different (≈39%, p<0.001). Although all methods provided values within the limits of agreement, the speed-based method provided the closest values to the "actual efficiency" | 6.0±0.0 |
| Stosic et al., (2021) | Examine the role of segmental, kinematic and coordinative parameters on the swimming velocity during the pre-transition and transition phases | 30 national level male swimmers (16.80±1.44 years) with a personal best time in the 100 m front crawl, backstroke, butterfly or breaststroke events within the 85% of world record (86.03±2.34%) | These results suggest that the body position and coordinative swimming parameters (apart from kicking or stroking rate and length) have an important influence on the transition performance, which depends on the swimming strokes and directly influences D_a_ and C_d_ | 6.0±0.0 |
| Takagi et al., (1999) | Measure D_a_, which is dynamic drag acting on a self-propelling swimmer in water | 4 collegiate skilled swimmers (21.0±0.8) | Was possible to measure D_a_ more precisely than before, and to obtain the experimental equation that predicted the D_a_ within a range of Reynolds number equaled the actual swimming velocity | 5.5±0.7 |
| Toussaint et al., (1988a) | The propelling efficiency (ep) of front-crawl swimming, by use of the arms only, was calculated. To measure mechanical power (P_o_) and drag (D_a_) by using a technique developed in laboratory. To report for the first time experimental determinations of ep in swimming humans | 4 male swimmers competing at international and national levels (mean: 22.3 years and 53.8 time of 100 m front crawl) | ep = D_a_/(D + P_k_*)=D_a_ / P_o_.  ep was found to range from 46 to 77%. Total efficiency, defined as the product of mechanical and propelling efficiency, ranged from 5 to 8%.  * kinetic energy of masses of water (P_k_) | 6.0±0.0 |
| Toussaint et al., (1988b) | Analyze D_a_ related to velocity in male and female swimmers. Propulsive arm forces were measured during front crawl swimming using arms only | 32 male and 9 female swimmers of good competitive level | It was found that D_a_ force is related to the swimming velocity v raised to the power 2.12±0.20 (males) or 2.28±0.35 (females). Differences in drag force and coefficient of drag between males and females (drag: 28.9±5.1 N, 20.4±1.9 N, drag coefficient: 0.64±0.09, 0.54±0.07 respectively) are especially apparent at the lowest swimming velocity (1 m/s), which become less at higher swimming velocities | 6.0±0.0 |
| Toussaint et al., (2002) | Study the effect on drag of a Speedo Fast‐skin suit compared to a conventional suit. The total D_a_ when swimming in the body Fastskin^TM^ was compared with that evoked when wearing conventional swimwear. | 13 subjects (6 males, 7 females) swimming at different velocities between 1.0 and 2.0 m/s | The D_a_ force was directly measured during front crawl swimming using a MAD system. For a range of swimming speeds (1.1, 1.3, 1.5 and 1.7 m/s). On a group level, a statistically non‐significant drag reduction effect of 2% was observed for the Fastskin^TM^ suit (p=0.31). Therefore, the 7.5% reduction in drag claimed by the swimwear manufacturer was not corroborated | 6.0±0.0 |
| Toussaint et al., (2004) | Determine whether the MAD and VPM system measure D_a_ | 6 top-level international competitive swimmers from the swimming team TZA (20.7±3.7). The mean performance for the 100m time(s) was 52.0±2.1 | The average drag for the VPM tests (53.2 N) was statistically significant and different from the D_a_ for the MAD-test (66.9 N). The regression of the relative difference in force (MAD vs VPM) on the relative difference in power was: %Deltadrag=1.898 x %Deltapower -4.498, R^2^=0.88. This suggests that the major part of the difference in D_a_ values is due to a non-equal power output in the 'free' relative towing trial during the VPM-test | 6.0±0.0 |
| Toussaint & Vervoorn, (1990) | Describe a new training device derived from the MAD system for front crawl swimming | 11 (8 male and 3 female, 18.50±3.30); Training group: 11 (8 male and 3 female, 18.40±2.10) | Despite the fact that training time and volume were equal, the training group showed a significantly greater improvement in force (from 91 to 94 N, 3.3%), velocity (from 1.75 to 1.81 m/s; 3.4%) and power (from 160 to 172 W, 7%) as measured on the MAD system, and an increase in distance per stroke in free swimming. | 6.0±0.0 |
| Van der vaart et al., (1987) | Calculate the drag on moving swimmers from direct propulsive arm force measurements (MAD System) | 12 male swimmers | The mean propulsive force at a velocity of v=1.48 m/s was shown to be 53.2±5.8 N (two to three times smaller than other studies for D_a_) which is in agreement with the values reported for D_p_ in a swimmer (towed on MAD System) that is not moving. In this study, discrepancies in D_a_ measurements are discussed | 5.0±0.0 |
| Vilas-Boas et al., (2010) | Assess and to compare the hydrodynamics of the first and second gliding positions of the breaststroke underwater stroke used after starts and turns | 12 national-level swimmers (6 males and 6 females, respectively 18.2±4.0 and 17.3±3.0 years) | For the same gliding velocities (1.37±0.124 m/s), Drag force and the swimmers' cross-sectional area and C_d_ values obtained for the first gliding position are significantly lower than the corresponding values obtained for the second gliding position of the breaststroke underwater stroke (31.67±6.44 N vs. 46.25±7.22 N; 740.42±101.89 cm^2^ vs. 784.25±99.62 cm^2^ and 0.458±0.076 vs. 0.664±0.234, respectively) | 6.0±0.0 |
| Xin-Feng et al., (2007) | Develop a simple and convenient device to measure the D_a_ at maximal velocity based on the equal power output assumption | 6 swimmers of national standard (3 males, 3 females) | For the males, the mean D_a_ ranged from 48.57 to 105.88 N in the front crawl and from 54.14 to 76.37 N in the breaststroke. For the females, the mean D_a_ ranged from 36.31 to 50.27 N in the front crawl and from 36.25 to 77.01 N in the breaststroke. The device provides a useful method for measuring and studying D_a_. | 6.0±0.0 |
| Zamparo et al., (1996) | Investigate whether the observed increases of unit of distance with underwater torque during front crawl swimming were due to an increase of D_a_, a decrease of drag efficiency or both | 8 male elite swimmers (21.1±1.36 years) at two submaximal speeds (1.00 and 1.23 m/s) | Underwater torque increased by 73% and that unit of distance, D_a_ and drag efficiency increased linearly with underwater torque. The increase of unit of distance between the two extremes was intermediate (≈20%) between that of D_a_ (≈35%) and of drag efficiency (≈16%). Thus, the actual strategy implemented by the swimmers to counteract underwater torque, was to tolerate a large increase of D_a_ | 5.0±0.0 |
| Zamparo et al., (2009) | Investigate the role of trunk incline and projected frontal area in determining drag during active/passive measurements | 6 elite college US swimmers (20.0±1.3 years)  25 subjects (14 male and 11 female swimmers, respectively 23.9±2.4 and 22.5±2.2 years) | Both projected frontal area and trunk incline were found to decrease with the swimming speed (D_a_/v^2^=67.7−22.2 × v, R=0.447, n=60, p<0.001) whereas the drag coefficient was found to be unaffected by the swimming speed. These data suggest that speed specific drag depend essentially on aprojected frontal area. This suggest, at a given speed, that D_a_ is larger than D_p_ | 6.0±0.0 |

D_a_ – active drag; D_p_ – passive drag; C_d_ – coefficient of drag; CD_a_ – coefficient of active drag; IdC – index of coordination; F_r_ – froude number; *F_d_* – active drag force; C_x_ – hydrodynamic coefficient; P_ai_ – metabolic power (power input); P_k_ - mechanical power to transfer; AP – pitch angle; ICC - intra-class correlation coefficients; SL—stroke length; SF - stroke frequency; SI—stroke index; SE - stroke efficiency; WS – whole stroke; dv—speed fluctuation; AS—arm span; IVV – intra-cyclic velocity variations; TDI – technique drag index; SPH – coupled biomechanical-smoothed particle hydrodynamics; FSA - frontal surface area; TTSA – trunk transverse surface; MAD – measuring active drag; ATM – assisted towing method; MRT – residual thrust measured values; VPM – speed perturbation method; AIS – assisted towing method; SD – one standard deviation.
